# Music that is used while studying and music that is used for sleep share similar musical features, genres and subgroups

**DOI:** 10.1038/s41598-023-31692-8

**Published:** 2023-03-23

**Authors:** Rebecca Jane Scarratt, Ole Adrian Heggli, Peter Vuust, Makiko Sadakata

**Affiliations:** 1 Department of Clinical Medicine, Center for Music in the Brain, Aarhus University and The Royal Academy of Music Aarhus/Aalborg, Aarhus, Denmark; 2 Music Department, The Netherlands and Institute for Logic, Language and Computation, University of Amsterdam, Amsterdam, The Netherlands

**Keywords:** Psychology, Human behaviour

## Abstract

Music is an integral part of daily human life, and certain types of music are often associated with certain contexts, such as specific music for sleeping or for studying. The mood-arousal hypothesis suggests that music used for studying should be uplifting to boost arousal and increase cognitive performance while previous studies suggest that music used as a sleep aid should be calm, gentle and slow to decrease arousal. In this study, we created the Study music dataset by collecting tracks from Spotify playlists with the words ‘study’ or ‘studying’ in the title or description. In comparison with a pre-existing dataset, the Sleep music dataset, we show that the music’s audio features, as defined by Spotify, are highly similar. Additionally, they share most of the same genres and have similar subgroups after a k-means clustering analysis. We suggest that both sleep music and study music aim to create a pleasant but not too disturbing auditory environment, which enables one to focus on studying and to lower arousal for sleeping. Using large Spotify-based datasets, we were able to uncover similarities between music used in two different contexts one would expect to be different.

## Introduction

Music plays a significant role in human life and is present in today’s society, in both work and leisure settings^1,2^. Music listening engages brain networks involved in hearing, motion, emotion and learning^3^, thereby modulating emotions, mood and arousal^4^. Many people use music to accompany behaviours, by modulating arousal either to relax as in the case of sleep aid music, or to stimulate the nervous system as in the case of music that is used while studying^5–9^. Thanks to the availability of large playlist data via online streaming services, identifying what music people use to accompany their daily activities has become increasingly popular among music scientists^10–13^. This leads to the discover of new music listening habits that reflect how the general population uses music. For example, whereas clinical trials usually use slow, soft and instrumental music to help relaxation and sleep^14–16^, recent studies are showing that people use much more varied music to help initiate sleep^12,17–19^. Additionally, while background music for studying is usually recommended to be calm and non-complex^20–22^, a recent study showed that how respondents used music was very varied^23^. These cases illustrate how music is used in practice may vary from how it was assumed in theory. By analysing multiple activity types together and using big data, we hope to gain more insight into the nature of the variation and elaborate on how music is used during those activity types in practice. In the present explorative study, we will compare music used for sleep and music used while studying. We think that sleep and study music are excellent domains to start with because the two activities are hypothesised to differ in their optimal arousal levels. While music is supposed to lower arousal for sleep, music is also supposed to increase arousal for better concentration while studying. However, in theory, both types of music should have similar musical features, such as calm and non-complex tracks^14–16,20–22^. Any similarity between the two would be informative to investigate general patterns in background music listening behaviours. Furthermore, we hope to show a new way to explore our musical behaviour using a data-driven approach: big data can provide insight into how music is used in reality, which could be different from theories.

The first type of music we will investigate is music used for sleep. As sleep problems increase worldwide so do self-help strategies, such as listening to music, to help fall asleep or to improve sleep quality^7,24–26^. Studies show that up to 46% of respondents indicate that they use music to help themselves fall asleep^7,27,28^ and that listening to music before sleep improves sleep quality across adult populations^6,9^.

The second type of music we will investigate is music that is used while studying. This type of music experienced a surge in popularity after the introduction of the so-called ‘Mozart Effect’, the effect of listening to music that temporarily enhances cognitive performance^29^. The original study proposed that listening to Mozart’s sonata K488 increased performance on a spatial cognitive task included in an IQ test^29^. Nowadays, another explanation, the mood-arousal hypothesis, is more commonly accepted to account for the increased cognitive performance while listening to music. This hypothesis proposes that the effect is caused by an increase in arousal attributed to the pleasant music. In other words, it proposes mood as a mediator of increased cognitive performance instead of the mystic power of classical music^8,30^. Many students utilise this mood-boosting function of music while studying which supposedly should benefit learning^20,31–35^. However, it is not easy to generalise about which type of music is most suitable for studying purposes. In general, music with low complexity—that has no words, a stable tonality and minimal changes in tempo and amplitude—seems to lead to the best effects on cognitive performance^22,36–43^. This is consistent with EEG brain studies showing that the auditory system responds strongly to pattern deviations^44^, a process which behaviourally may result in an orienting response and ultimately that the listener gets distracted from studying^45^. The mood-arousal hypothesis predicts that pleasant and simple music induces the mood-boosting effect while unpleasant or too arousing music hinders the benefit. Just like how there is an inverted U-shape for groove preferences^46^ and how the Yerkes–Dodson law sees an inverted U-shape model as the link between arousal and performance, with optimal performance at moderate arousal levels^47^, there seems to be an inverted U-shape modelling the complexity of a piece of music and the mood-boosting effect. It appears that the type and difficulty of the task as well as individual differences, interact significantly with the beneficial effect of background music listening while studying, making it challenging to draw a simple answer^23^.

A recent survey with 140 participants shows that the music people listen to while studying varies considerably, such as non-vocal, vocal, calm, jazz, pop, classic and upbeat music^23^. Interestingly, these genres largely overlap with those present in the music playlists people use as sleep aids, based on a big data study of Spotify playlists^12^. Does this entail that the music used for sleep is similar to the music used for studying? Sleeping and studying involve different cognitive processes, and the desired mood and status for these activities are also different: Sleep music induces relaxation, helping the sleep process, whereas Study music induces optimal levels of arousal while not distracting the studying listener, helping cognitive performance. It would be puzzling, however, if the same type of music would be used to accompany two almost opposite activities, but it also opens new research possibilities, giving further insights into the interaction between the type of music, task, and individual differences in the effective use of background music.

This study is focused on determining to which extent the characteristics of music used for sleep and study are shared, and on what parameters they are similar or dissimilar. Firstly, we form a dataset of music for studying collected from publicly available Spotify playlists. Secondly, we compare three datasets to establish the differences and similarities among them: the newly formed Study playlist dataset, the Sleep playlist dataset^12^ and the Music Streaming Session dataset^48^, which is used as a proxy for General music. In doing so, we shed light on human behaviour surrounding music listening and on arousal modulation which can be used to advise music-based interventions.

## Methods

In order to assess the similarities between sleep music and study music, we used a pre-existing dataset of sleep music, we formed a new dataset of study music, and we used a pre-existing general music dataset as a control. These datasets were compared qualitatively on a track level, a genre level, and on a cluster level. They were also compared quantitatively by individual audio features, and by the statistical distance between their audio features. After introducing each dataset, the methods for each analysis will be described below.

### The datasets

#### The Study music dataset

To build the global dataset of Study music, we automatically searched the online streaming service Spotify for playlists that contained the words ‘study’ or ‘studying’ in the title or the description using the Python package GSA^49^ set to automatically exclude playlists with less than 50 followers. These titles and descriptions are added by the creators of the playlists, which can either be Spotify users or Spotify itself. They usually reflect how the creator intends the playlist to be used, for example ‘Focus-enhancing piano for your study session’. The search was performed in spring 2021 and yielded 801 playlists. As the search was done systematically but automatically, the playlist titles were inspected manually to verify the word ‘study’ was used to describe the act of listening to music while studying. Eleven playlists were excluded as they included different uses of the word study, such as ‘John Hopkins Psilocybin study’, or if they were not music such as ‘White Noise for Studying’. The dataset contains 172,819 tracks spread over 790 playlists. Of these tracks, 63,418 tracks appear in the dataset more than once, meaning there are 109,628 unique tracks in this dataset. With the Python package GSA^49^, we were able to retrieve 9 audio features measured by Spotify (see Table 1), as well as the tracks’ Spotify trackID, the playlists’ Spotify ID and the compound genre tag (see Genre Reduction section for more details on genre).

**Table 1:** Overview of the audio features that are accessible through the Spotify API and their descriptions as given by Spotify^50^.

Audio feature	Description
Acousticness	A confidence value indicating how likely a track is acoustic, meaning performed on non-amplified instruments, ranging between 0 and 1
Danceability	A value indicating how suitable a track is for dancing, ranging between 0 and 1, with higher values indicating increased danceability
Energy	A value indicating a perceptual measure of intensity and activity, ranging between 0 and 1
Instrumentalness	A confidence value indicating how likely a track contains no vocals, ranging between 0 and 1 with values above 0.5 likely to be instrumental tracks
Liveness	A confidence value indicating how likely a track is performed live, for instance by detecting the sound of an audience in a recording
Loudness	A value indicating the overall loudness of a track, ranging between − 60 and 0 dB. Spotify does not specify a dB scale, but it is assumed this is measured in LUFS or a similar perceptual loudness scale
Speechiness	A value indicating the presence of spoken words in a track
Tempo	A value indicating the speed or pace of track, as estimated by the average beat duration, given in beats-per-minute (BPM)
Valence	A value indicating positively valenced a track is (from a Western point of view), ranging between 0 and 1, with high values indicated a track that is likely to be perceived as more positive, happy, and cheerful

#### The Sleep music dataset

The Sleep playlists dataset was created by extracting 225,626 tracks from 985 playlists including the words ‘Sleep’, ‘Sleepy’ or ‘Sleeping’ in the title or description^12^. Of these tracks, 130,150 are unique. The dataset contains the same nine audio features as the Study music dataset, the track and playlist IDs as well as genre tags.

The Study and Sleep datasets share 21,872 tracks. These tracks are mostly *lo-fi and pop* tracks.

#### The General music dataset

The Music Streaming Session dataset is a publicly available dataset released by Spotify on CrowdAI^48^ and contains the audio features of approximately 3.7 million unique tracks that were collected over multiple weeks in 2019. The tracks were listened to at all hours of the day by people across the world. We treat this dataset as a proxy for General music listening as the tracks were collected over different regions, people and times without reflecting a specific behaviour other than ‘music listening’. This dataset contains the same 9 audio features measured by Spotify as the previous two datasets but does not contain the tracks’ Spotify trackID, the playlists’ Spotify IDs or genre tags.

### Qualitative comparison

#### Comparing genres between the datasets

All Spotify tracks are assigned a genre tag, such as “Icelandic post-punk” or “Instrumental maths rock”, and in most cases one single track has multiple genre tags. In order to understand the data better, we applied a genre reduction algorithm^51^. This algorithm aims to reduce the list of sub-genres provided by Spotify for a particular track such that: $G\left( x \right) = argmax_{y} \left( {\sum\nolimits_{i = 1}^{n} {g_{y} \left( {x_{i} } \right)} } \right)$, where $x$ is the list of sub-genres of a track, and $G\left( x \right)$ is the main genre, obtained by calculating whether each predetermined main genre $y$ is a substring of the sub-genre $x_{i}$, and then choosing the main genre with the most occurrences.

A Python-implementation is available at GitHub.com/RebeccaJaneScarratt/Study-Sleep-Analyses. The list of main genres were created from a 14-item scale assessing preferences in music genres: the Short Test Of Music Personality (STOMP) scale^52^. We replaced Oldies with 5 additional genres that were added in Trahan et al.^17^ and added 4 genres ourselves. The 4 additional genres were chosen due to their high prevalence in the dataset. They stem from the genres that Spotify assigns to its tracks such as “soft sleep chill”. These genres are assigned based on information from listener playlists and the Spotify music curation team^53^. For a full overview of the 31 genres see Table 2.

**Table 2:** All main genres used to reduce the genre tags.

Origin	Genres
STOMP Genres	Alternative, Bluegrass, Blues, Christian, Classical, Country, Electronic, Folk, Funk, Gospel, Jazz, Metal, New age, Opera, Pop, Punk, R&B, Rap, Reggae, Rock, Soundtrack, World
From Trahan et al.^17^	Ambient, House, Indie, Instrumental, Meditation
Additional genres	Background, Lo-fi, Lullaby, Sleep

#### Comparing subgroups between the Sleep and Study music

In previous work on the Sleep Playlist dataset, a large variation of genres and audio features within the dataset was found. Subsequently, a k-means clustering analysis was performed on the audio features and revealed 6 distinct clusters. To assess similar variation in the unique Study dataset, we performed the same k-means clustering on the audio features in the same way as in Scarratt et al. (2021). This was performed in RStudio using its inbuilt k-means function, which divides the data into a certain number of clusters, k, by minimising within-cluster variance. A maximum of 1000 iterations were used. To select the optimal k, we used the elbow-method^54^. In our case, this resulted in an optimal division of the unique Study audio feature data into 3 clusters. These subgroups were then compared qualitatively using a radar plot visualization with the medians of each audio features per cluster. To include them in the radar plots, Loudness and Tempo were normalised.

### Quantitative comparison

#### Comparing individual audio features between the datasets

To compare differences between the datasets, we used the nine audio features available from Spotify. These cover a wide range of both basic and compound musical measures. As the calculation of these audio features are proprietary, and we were unable to quantify exactly which calculations and transformations underlie each feature, we based our interpretation of the audio features on Spotify’s description as part of their Application Programming Interface (API) reference manual^50^ (see Table 1). The comparison of the individual audio features between the datasets was done using t-tests with Welsch correction, with Cohen's D as a measure of effect size. The audio features Instrumentalness and Acousticness exhibited a strong bimodal distribution. Therefore, we dichotomised these values with a split point at 0.5, and calculated statistical difference using the Chi-Square test, with Cramêr’s V as a measure of effect size. All *p* values were Bonferroni corrected.

#### Comparing statistical distance between the datasets

To gain an overlying measure of distance between the three datasets, we used the Kolomogorov-Smirnov (KS) distance statistic^55^. This value increases with the maximum distance between two sample’s empirical distribution function. Here we calculated the KS statistic for each of the audio features Danceability, Energy, Loudness, Speechiness, Acousticness, Instrumentalness, Liveness, Valence, and Tempo, between the three possible comparisons of datasets. To interpret the results, we then took the mean of each comparisons’ individual audio feature KS statistics, giving us one value for each comparison.

All statistical analyses were performed in RStudio version 1.3.959 using R version 4.0.0, running on Windows 10. The scripts used for analysing the dataset can be found at GitHub.com/OleAd/SpotifyStudyMusic. Figures were made using ggplot2 and the RainCloudPlots package^56^ or the Python packages Matplotlib and Plotly. The genre reduction script was performed in Spyder using Python version 3.9.

## Results

As detailed in the methods section, to assess the similarities between sleep music and study music, we compared a Study music dataset with a Sleep music dataset and a General music dataset as a control. They were compared using qualitative and quantitative methods.

### Qualitative comparison

#### Comparing genres between the datasets

The genres that appear more than 1000 times in either the unique Sleep music dataset or unique Study music dataset are listed in Table 3 to compare the prevalence of each genre in both datasets. As the Spotify API is not always able to identify the genre of a track, 29,274 tracks from the Study dataset and 32,885 tracks from the Sleep dataset were excluded from this genre analysis.

**Table 3:** Genres that appear more than 1000 times in either the unique Sleep music dataset or that appeared more than 1000 times in the unique Study music dataset sorted in descending order according to their prevalence in the Study dataset.

Genre	Study unique tracks (N = 109,628) Number of occurrences (percentage in the unique dataset)	Sleep unique tracks (N = 130,150) Number of occurrences (percentage in the unique dataset)
Pop	15,113 (13.79%)	14,121 (10.85%)
Lo-fi	13,048 (11.90%)	6149 (4.72%)
Classical	7828 (7.14%)	3907 (3.00%)
Soundtrack	7109 (6.48%)	2028 (1.56%)
Instrumental	3547 (3.23%)	3275 (2.52%)
Jazz	2969 (2.71%)	4167 (3.20%)
House	2822 (2.57%)	< 1000 (< 1%)
Sleep	2319 (2.11%)	18,730 (14.40%)
Rap	2160 (1.97%)	4444 (3.42%)
Ambient	1923 (1.75%)	7466 (5.74%)
Indie	1716 (1.56%)	2101 (1.61%)
Rock	1639 (1.50%)	2550 (1.96%)
Background	1073 (< 1%)	1970 (1.51%)
Electronic	1064 (< 1%)	< 1000 (< 1%)
Lullaby	< 1000 (< 1%)	3519 (2.70%)
Meditation	< 1000 (< 1%)	2006 (1.54%)
Folk	< 1000 (< 1%)	1520 (1.17%)
Country	< 1000 (< 1%)	1172 (< 1%)
Christian	< 1000 (< 1%)	1117 (< 1%)
World	< 1000 (< 1%)	1093 (< 1%)
R&B	< 1000 (< 1%)	1052 (< 1%)

Many tracks of each dataset, 10,792 tracks of Study music (9.4%) and 11,624 (8.9%) tracks of Sleep music, were unable to be categorised due to their obscure genre labels. Furthermore, there were 782 uncategorized genres in the Study dataset and 789 in the Sleep dataset. These genres, such as ‘otacore’ or ‘dangdut’, were not able to be matched to any of the genre categories defined in Table 2. There were relatively few of each of these genres, with the most prevalent Study uncategorised genre being ‘chillhop’ with 722 occurrences and ‘drift’ as the most prevalent uncategorised Sleep genre with 545 occurrences, meaning that their inclusion as a new genre would not have altered this table.

#### Comparing subgroups between the Sleep and Study datasets

##### Subgroup characteristics of Study music

When performing a k-means clustering analysis, the Study music dataset was found to have three distinct clusters. Cluster 1 in Table 4 (N = 35,729) is characterised by low Acousticness (M = 0.35), medium Instrumentalness (M = 0.46), medium Energy (M = 0.49) and high Danceability (M = 0.66). Because many of the tracks in this cluster are classified by Spotify as *pop* (N = 10,765), it was named ‘Pop Tracks’. Cluster 2 (N = 34,617) is characterised by high Acousticness (M = 0.952), high Instrumentalness (M = 0.902), low Energy (M = 0.132) and medium Danceability (M = 0.403). Many of the tracks are *classical* (N = 6296) and *soundtrack* (N = 5852), and therefore it was named ‘Classical Soundtrack Tracks’. Cluster 3 (N = 39,282) is characterised by low Acousticness (M = 0.271), low Instrumentalness (M = 0.196), medium Energy (M = 0.57) and high Danceability (M = 0.61) and is mainly composed of *lo-fi* tracks (N = 9468), and therefore it was named ‘Lo-fi Tracks’ (Table 4).

**Table 4:** Medians and interquartile ranges of all audio features for the 3 clusters of the Study dataset.

		N	Danceability	Energy	Loudness (db)	Speechiness	Acousticness	Instrumentalness	Liveness	Valence	Tempo (bpm)
Cluster 1	Pop Tracks	35,729	0.66 (0.19)	0.49 (0.30)	− 9.17 (4.85)	0.05 (0.06)	0.35 (0.60)	0.46 (0.86)	0.11 (0.04)	0.42 (0.36)	117.0 (49.7)
Cluster 2	Classical Soundtrack Tracks	34,617	0.40 (0.31)	0.13 (0.18)	− 19.56 (8.38)	0.04 (0.02)	0.95 (0.12)	0.90 (0.10)	0.11 (0.03)	0.15 (0.21)	98.8 (52.0)
Cluster 3	Lo-fi Tracks	39,282	0.61 (0.20)	0.57 (0.35)	− 8.65 (5.89)	0.06 (0.08)	0.27 (0.62)	0.20 (0.83)	0.37 (0.19)	0.42 (0.38)	115.6 (51.1)

##### Subgroup characteristics of Sleep music

A k-means clustering analysis was performed on Sleep music in a previous study^12^. The following six clusters were identified: ‘Speechy Tracks’, ‘Radio Tracks’, ‘Acoustic Radio Tracks’, ‘Ambient Tracks’, ‘Instrumental Tracks’ and ‘Live Tracks’ (Table 5). The two biggest clusters were ‘Ambient Tracks’ (N = 117,240) which contained instrumental, ambient, meditation music and ‘Instrumental Tracks’ (N = 32,736), containing instrumental pieces and instrumental covers. They were both characterised by high Acousticness (M = 0.957 and M = 0.888 respectively), high Instrumentalness (M = 0.917 and M = 0.893) and low Energy (M = 0.0423 and M = 0.172). ‘Instrumental Tracks’ had higher Danceability (M = 0.655) than ‘Ambient Tracks’ (M = 0.207) as the instrumental music contained in the latter had a steadier pulse and beat compared to the ambient music from ‘Ambient Tracks’. Additionally, ‘Radio Tracks’ (N = 31,068) and ‘Acoustic Radio Tracks’ (N = 30,793) contained popular tracks that one could likely find on the radio, including pop, indie and soul music. They both had low Instrumentalness (M < 0.001 and M < 0.001), medium Energy (M = 0.597 and M = 0.278), high Danceability (M = 0.622 and M = 0.496) and low (M = 0.155) or high (M = 0.818) Acousticness respectively. These four subgroups are almost opposites and point towards two different behaviours when it comes to listening to music before sleep, either listening to soft, slow, instrumental tracks or listening to known non-instrumental music. The final two clusters either had high Speechiness (M = 0.334) compared to all other clusters (M < 0.06 in all other clusters), giving the ‘Speechy Tracks’ cluster (N = 8275), or high Liveness (M = 0.689), giving the ‘Live Tracks’ cluster (N = 5783).

**Table 5:** Medians of all audio features for the 6 clusters of the Sleep dataset.

		N	Danceability	Energy	Loudness (db)	Speechiness	Acousticness	Instrumentalness	Liveness	Valence	Tempo (bpm)
Cluster 1	Speechy Tracks	8275	0.68 (0.21)	0.45 (0.31)	− 10.01 (5.97)	0.33 (0.14)	0.50 (0.59)	< 0.01 (0.71)	0.12 (0.11)	0.46 (0.32)	102.0 (62.64)
Cluster 2	Radio Tracks	31,068	0.62 (0.20)	0.60 (0.22)	− 7.09 (3.62)	0.04 (0.04)	0.16 (0.28)	< 0.01 (0.08)	0.12 (0.08)	0.41 (0.32)	119.9 (46.1)
Cluster 3	Acoustic Radio Tracks	30,793	0.50 (0.20)	0.29 (0.19)	− 11.05 (5.5)	0.04 (0.01)	0.82 (0.23)	< 0.01 (0.007)	0.11 (0.04)	0.24 (0.2)	111.8 (46.4)
Cluster 4	Ambient Tracks	117,240	0.20 (0.15)	0.04 (0.10)	− 27.95 (10.4)	0.04 (0.01)	0.96 (0.10)	0.92 (0.09)	0.11 (0.03)	0.05 (0.08)	83.2 (50.4)
Cluster 5	Instrumental Tracks	32,736	0.66 (0.16)	0.18 (0.19)	− 17.44 (8.13)	0.05 (0.03)	0.89 (0.24)	0.89 (0.11)	0.11 (0.02)	0.40 (0.32)	92.5 (40.0)
Cluster 6	Live Tracks	5783	0.30 (0.21)	0.29 (0.38)	− 18.52 (15.4)	0.05 (0.03)	0.83 (0.47)	0.86 (0.88)	0.70 (0.26)	0.15 (0.26)	95.1 (43.0)

##### Comparing the sub-groups of the two datasets

**Figure 1:**
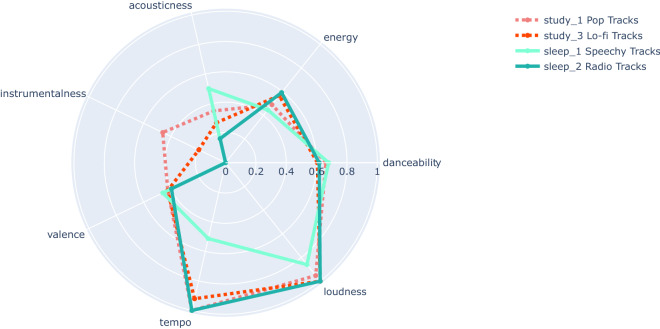
Radar plot of 7 audio features of Study clusters 1 (‘Pop Tracks’) and 3 (‘Lo-fi Tracks’) and Sleep clusters 1 (‘Speechy Tracks’) and 2 (‘Radio Tracks’).

**Figure 2:**
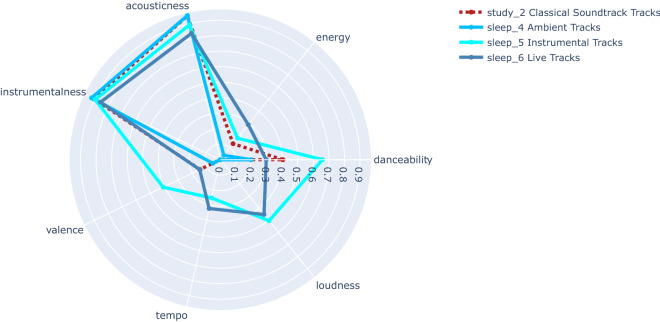
Radar plot of 7 audio features of Study cluster 2 (‘Classical Soundtrack Track’s) and Sleep clusters 4 (‘Ambient Tracks’), 5 (‘Instrumental Tracks’) and 6 (‘Live Tracks’).

Seven audio features of all the clusters from the Study and the Sleep datasets were represented in a radar plot that highlights similarities between clusters between datasets. There appeared to be three groups between the clusters of the different datasets. Study clusters 1 (‘Pop Tracks’) and 3 (‘Lo-fi Tracks’) share similar features to Sleep clusters 1 (‘Speechy Tracks’) and 2 (‘Radio Tracks). Study cluster 2 (‘Classical Soundtrack Tracks’) has similar features to Sleep clusters 4 (‘Ambient Tracks’), 5 (‘Instrumental Tracks’) and 6 (‘Live Tracks’). Sleep cluster 3 (‘Acoustic Radio Tracks’) does not share consistent similarities to other clusters. The similarities between clusters were highlighted by isolating the two first groups in separate plots (see Figs. 1, 2).

### Quantitative comparison

#### Comparing individual audio features between the datasets

**Figure 3:**
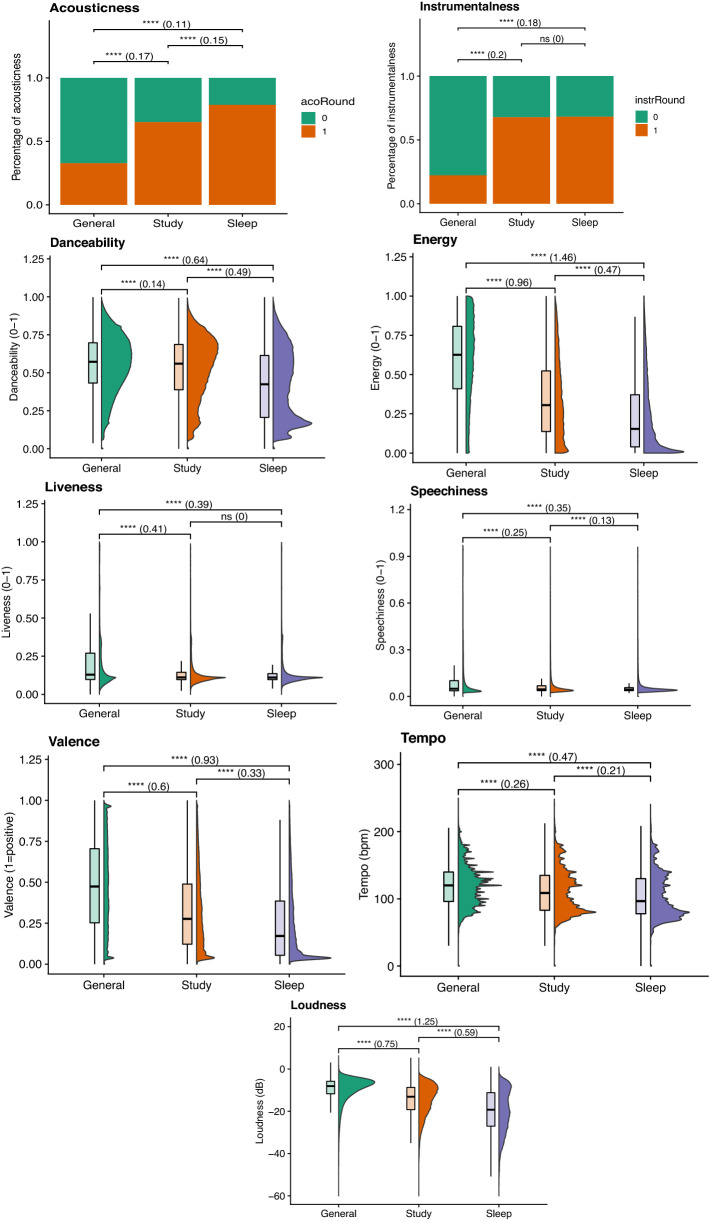
Density plots, t-test significance asterisks and Cramer’s V statistic in brackets between each pair of datasets for each audio feature for all audio features except for Acousticness and Instrumentalness where a chi-squared test was used. ****, *p* < 0.001 Bonferroni corrected.

The density plots of each audio feature per dataset can be found in Fig. 3. The results of the pairwise t-tests are represented by significance asterisks. Generally, all the comparisons are significant, except for Instrumentalness and Liveness where there is no significant difference between the Study and Sleep datasets. Due to the size of the datasets, it is expected to find many significant comparisons even after correcting for multiple comparisons. Therefore, we primarily interpret the effect size, Cohen’s D, for all audio features except Instrumentalness and Acousticness where Cramer’s V was used. Cramer’s V was interpreted using the following categories: < 0.20 = negligeable, 0.20–0.50 = Small, 0.50–0.90 = Moderate, > 0.90 = Large. The large effect sizes between the Sleep and General datasets are in Loudness (*p* < 0.001, d = 1.25, Energy (*p* < 0.001, d = 1.46) and Valence (*p* < 0.001, d = 0.93) as well as between the General and Study datasets in Energy (*p* < 0.001, d = 0.96), Loudness (*p* < 0.001, d = 0.75) and Valence (*p* < 0.001, d = 0.60). There were no large effect sizes between the Study and Sleep datasets, only a moderate effect size in Loudness (*p* < 0.001, d = 0.59).

#### Comparing statistical distance between the datasets

**Figure 4:**
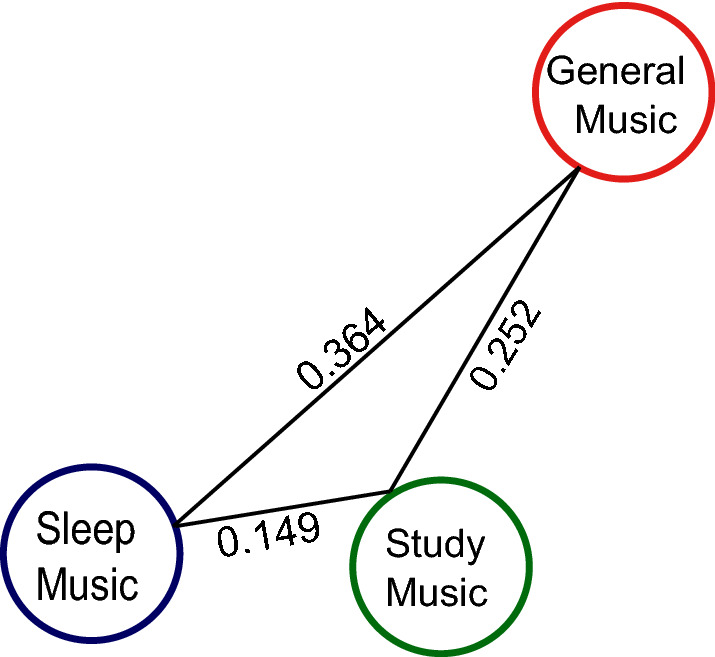
The Kolmogorov–Smirnov distance between General music and Sleep music, Sleep music and Study music and between General music and Study music across all nine audio features (Instrumentalness, Acousticness, Speechiness, Liveness, Tempo, Loudness, Energy, Valence and Danceability).

When comparing the three datasets pairwise to each other with the Kolmogorov–Smirnov distance, we found that Study music is more similar to Sleep music (0.149) than to General music (0.364). Sleep music is more similar to Study music (0.149) than to General music (0.252). However, Study music is more similar to General music (0.252) than Sleep music is to General music (0.364; see Fig. 4).

## Discussion

Using both qualitative and quantitative analyses based on tracks, genres or audio features, we aimed to exploratively compare Study music and Sleep music. Using datasets generated from Spotify’s database, we found that Sleep music and Study music were more similar to each other than to General music (see Fig. 4). Furthermore, Sleep and Study music share a similar distribution in all audio features, do not differ in Liveness or Instrumentalness, include similar genres and have similar subgroups.

Furthermore, the two datasets also included music from similar genres. Out of the 12 top genres in the Study playlist dataset (*pop, lo-fi, classical, soundtrack, instrumental, jazz, house, sleep, rap, ambient, indie and rock*), every genre was present in the Sleep playlists dataset except *house.* The Study playlist dataset also included 2319 tracks with the genre *sleep* (a genre that is named as such by Spotify and includes tracks that the creators of the track deemed as relevant for sleep), an overt demonstration that Study music shares similarities with Sleep music. Additionally, 21,872 unique tracks appeared both in the Sleep playlist dataset and in the Study dataset, indicating that many tracks are equally suited for sleeping and studying.

Our cluster analyses (Figs. 1, 2) demonstrated similarities in the characteristics of clusters between the Study and Sleep music datasets. Specifically, there seems to be three groups. One group contains ‘Speechy’, ‘Radio’, ‘Pop’ and ‘Lo-fi’ tracks, characterised by high Danceability, high Energy, medium–low Instrumentalness and Acousticness. Another group contains ‘Ambient, ‘Instrumental’, ‘Live’ and ‘Classical’ tracks, characterised by high Instrumentalness and Acousticness and medium–low Danceability and Energy, and finally one group has ‘Acoustic Radio Tracks’ and is characterised by high Acousticness, low Instrumentalness, low Energy and medium Danceability. We interpret that these different groupings of audio tracks reflect different preferences, given that each track was added to these datasets based on human action. The group of ‘Speechy’, ‘Radio’, ‘Pop’ and ‘Lo-fi’ tracks suggests a preference of certain individuals to listen to and therefore add these types of tracks to sleep or study playlists, whereas another preference is to listen to ‘Acoustic Radio Tracks’. Some individuals might like to swap music types frequently e.g., to find their optimal background music. However, we think that once established, an individual would stick to similar sounding tracks for a certain activity. Thus, we argue that these three different groupings suggest three separate listening habits.

When looking into the audio features, we found that the Instrumentalness and Liveness in Study and Sleep music were comparable and, although other features are statistically different between the two datasets, the effect size tended to be rather small. The characteristics of General music were more distanced to those of Sleep and Study music when all three were compared. General music is characterised by being more energetic, happier and louder than Sleep music and Study music.

A crucial question is why there is such an overlap between Study music and Sleep music? Given the very different nature of activities involved in sleep and study, it is surprising that people use similar types of music to accompany these tasks. Curiously, there might be a similarity in why people use music for Sleep and Study. For sleep, one of the most popular motivations is to distract one from thoughts that might disrupt sleep^17^. For studying, setting a good mood and helping concentration are popular motivations for using music^23^. In other words, in both activities, people use music to create a pleasant auditory environment and focus on a specific task. To do so, accompanying music should not attract too much attention as this will decrease performance^21,45^. Therefore, these two datasets might both contain music with the optimal stimulation amounts in order to create a suitable pleasant auditory environment.

Indeed, the shared features between Sleep and Study music are lower values of Valence, Energy, Tempo, Loudness, and higher values of Acousticness and Instrumentalness. In other words, both datasets included more music tracks that are less loud, less fast and instrumental than the General music playlist. Our typical responses for slow tempo non-vocal music are lower arousal, physiological responses and somewhat negative mood^57–59^.

However, everyone does not react to music in the same way. Age, culture, personality, musical expertise and musical familiarity might all influence how a certain individual reacts to a piece of music^60–66^. These individual differences might explain the intra-dataset variation in both Study music and Sleep music datasets. For example, individuals with high extraversion are said to require more stimulation before reaching the ideal arousal level whereas individuals with high introversion require less stimulation to reach the same arousal level^61,67^. Therefore, it is highly likely that individual differences, such as personality traits and other factors, influence the choice of music to accompany people’s sleep and study. Such individual differences may be reflected in the various subgroups found for Sleep and Study music (see Figs. 1, 2). Investigating which specific individuals choose to listen to while studying or going to sleep could help understand which traits (personality, age, culture…) influence music choices related to studying and sleeping.

The current study highlights a new study research trend which uses large datasets acquired from Spotify, namely a comparison of music used to study and to sleep. Having such a large dataset is beneficial for statistical power and general level analyses that would have been difficult to conduct otherwise. Because of company politics, Spotify does not provide any demographic information, which means that the present datasets do not allow us to investigate who is listening to the playlists. Therefore, we cannot rule out a potential mismatch between the groups of the listeners. For instance, most people listening to Study playlists may be students within a certain age range while those listening to Sleep playlists may reflect a different age range. Specifically, the large prevalence of Lo-fi in the Study playlists could reflect a younger population, due to Lo-fi’s relative recency as a genre. To better match and expand the people who are using the playlists, future studies could benefit from investigating music playlists listened to at work, using search terms like ‘office’ or ‘programming’.

Importantly, the overlap between the two datasets does not necessarily mean that the same person is using the same music tracks for both sleeping and studying. For drawing stronger associations between the Sleep and Study music as well as underlying cognitive mechanisms, interventional approaches (e.g., using smart watches), conventional experimental approaches, or a large-scale survey with more explicit demographic information could be considered, which would allow a look into the details of individual behaviours. Additionally, no information on how the playlists are used is available. While we know that the creator of the playlist had a specific behaviour in mind when creating the playlist, no conclusions can be drawn about how the followers or users of the playlists use them. However, it is likely that someone would use a playlist named ‘Music for Sleeping: Calm Piano Sleep Aid, Music for Relaxation And The Best Sleep Music’ to help themselves sleep and that a playlist called ‘Chill music mix to study to’ would be used while studying.

In conclusion, our results show that a similarity exists between Study music and Sleep music, in terms of genres, audio features, and in their comparison to General music. While it is not possible to give concrete explanation for this similarity from our study, we suggest that music creates a pleasant but not too disturbing atmosphere which enables one to focus on studying and to lower arousal for sleeping. Given the variety of genres and audio features within the playlists, it is clear that individual differences and preferences also come into play. The similarity of these two types of music in relation to their desired effect on arousal is surprising and suggests a re-evaluation of the role of arousal in studying, or of the influence of music on arousal. The exact influence of these music types on arousal is still unknown and should be investigated further to understand better the function of these music types. Nevertheless, our results highlight the benefit of using big data sets to compare different types of music, yielding strong evidence that music used for sleep and music used for studying, somewhat surprisingly, share many characteristics.

## Data and code availability

The full datasets and the code are available at https://github.com/RebeccaJaneScarratt/Study-Sleep-Analyses.

## References

[1] Greenberg DM, Rentfrow PJ (2017). Music and big data: A new frontier. Curr. Opin. Behav. Sci..

[2] North AC, Hargreaves DJ, Hargreaves JJ (2004). Uses of music in everyday life. Music Percept..

[3] Vuust P, Heggli OA, Friston KJ, Kringelbach ML (2022). Music in the brain. Nat. Rev. Neurosci..

[4] Sloboda, J. A. & O’neill, S. A. Emotions in everyday listening to music. *Music Emot. Theory Res.* 415–429 (2001).

[5] Dickson GT, Schubert E (2019). How does music aid sleep? Literature review. Sleep Med..

[6] Jespersen KV, Koenig J, Jennum P, Vuust P (2015). Music for insomnia in adults. Cochrane Database Syst. Rev..

[7] Morin CM, LeBlanc M, Daley M, Gregoire JP, Merette C (2006). Epidemiology of insomnia: Prevalence, self-help treatments, consultations, and determinants of help-seeking behaviors. Sleep Med..

[8] Nantais KM, Schellenberg EG (1999). The Mozart effect: An artifact of preference. Psychol. Sci..

[9] Wang C-F, Sun Y-L, Zang H-X (2014). Music therapy improves sleep quality in acute and chronic sleep disorders: A meta-analysis of 10 randomized studies. Int. J. Nurs. Stud..

[10] Heggli OA, Stupacher J, Vuust P (2021). Diurnal fluctuations in musical preference. R. Soc. Open Sci..

[11] Holtz, D. *et al. The Engagement-Diversity Connection: Evidence from a Field Experiment on Spotify*. https://papers.ssrn.com/abstract=3555927. (2020) 10.2139/ssrn.3555927 (2020).

[12] Scarratt RJ, Heggli OA, Vuust P, Jespersen KV (2022). The audio features of sleep music: Universal and subgroup characteristics. PLoS ONE.

[13] Tricahyono, D., Utami, L. W. & Safitri, W. The impact of viral marketing on consumers’ intention to use (case study: Spotify Indonesia). In 674–678 (Atlantis Press, 2019). 10.2991/icebef-18.2019.144

[14] Bernardi L (2005). Cardiovascular, cerebrovascular, and respiratory changes induced by different types of music in musicians and non-musicians: The importance of silence. Heart.

[15] Jespersen KV, Pando-Naude V, Koenig J, Jennum P, Vuust P (2022). Listening to music for insomnia in adults. Cochrane Database Syst. Rev..

[16] Gaston ET (1951). Dynamic music factors in mood change. Music Educ. J..

[17] Trahan T, Durrant SJ, Müllensiefen D, Williamson VJ (2018). The music that helps people sleep and the reasons they believe it works: A mixed methods analysis of online survey reports. PLoS ONE.

[18] Dickson GT, Schubert E (2020). Musical features that aid sleep. Music. Sci..

[19] Tan X, Yowler CJ, Super DM, Fratianne RB (2012). The interplay of preference, familiarity and psychophysical properties in defining relaxation music. J. Music Ther..

[20] Ransdell S, Gilroy L (2001). The effects of background music on word processed writing. Comput. Hum. Behav..

[21] Thompson WF, Schellenberg EG, Letnic AK (2012). Fast and loud background music disrupts reading comprehension. Psychol. Music.

[22] Dalton BH, Behm DG (2007). Effects of noise and music on human and task performance: A systematic review. Occup. Ergon..

[23] Goltz F, Sadakata M (2021). Do you listen to music while studying? A portrait of how people use music to optimize their cognitive performance. Acta Psychol. (Amst.).

[24] Calem M (2012). Increased prevalence of insomnia and changes in hypnotics use in England over 15 years: Analysis of the 1993, 2000, and 2007 National Psychiatric Morbidity Surveys. Sleep.

[25] Garland SN (2018). A decade’s difference: 10-year change in insomnia symptom prevalence in Canada depends on sociodemographics and health status. Sleep Health.

[26] Léger D, Poursain B, Neubauer D, Uchiyama M (2008). An international survey of sleeping problems in the general population. Curr. Med. Res. Opin..

[27] Brown CA, Qin P, Esmail S (2017). “Sleep? Maybe later…” A cross-campus survey of university students and sleep practices. Educ. Sci..

[28] Urponen H, Vuori I, Hasan J, Partinen M (1988). Self-evaluations of factors promoting and disturbing sleep: An epidemiological survey in Finland. Soc. Sci. Med..

[29] Rauscher FH, Shaw GL, Ky CN (1993). Music and spatial task performance. Nature.

[30] Chabris CF (1999). Prelude or requiem for the ‘Mozart effect’?. Nature.

[31] Alisaari J, Heikkola LM (2016). Increasing fluency in L2 writing with singing. Stud. Second Lang. Learn. Teach..

[32] Calderwood C, Ackerman PL, Conklin EM (2014). What else do college students “do” while studying? An investigation of multitasking. Comput. Educ..

[33] de Groot AMB (2006). Effects of stimulus characteristics and background music on foreign language vocabulary learning and forgetting. Lang. Learn..

[34] Johansson R, Holmqvist K, Mossberg F, Lindgren M (2012). Eye movements and reading comprehension while listening to preferred and non-preferred study music. Psychol. Music.

[35] Kiger DM (1989). Effects of music information load on a reading comprehension task. Percept. Mot. Skills.

[36] Jones M, West S, Estell D (2006). The Mozart effect: Arousal, preference, and spatial performance. Psychol. Aesthet. Creat. Arts.

[37] Schellenberg EG (2005). Music and cognitive abilities. Curr. Dir. Psychol. Sci..

[38] Schubert E (2007). The influence of emotion, locus of emotion and familiarity upon preference in music. Psychol. Music.

[39] Thompson WF, Schellenberg EG, Husain G (2001). Arousal, mood, and the Mozart effect. Psychol. Sci..

[40] Proverbio AM, De Benedetto F (2018). Auditory enhancement of visual memory encoding is driven by emotional content of the auditory material and mediated by superior frontal cortex. Biol. Psychol..

[41] Hallam S, Price J, Katsarou G (2002). The effects of background music on primary school pupils’ task performance. Educ. Stud..

[42] Angel LA, Polzella DJ, Elvers GC (2010). Background music and cognitive performance. Percept. Mot. Skills.

[43] Proverbio AM (2015). The effect of background music on episodic memory and autonomic responses: Listening to emotionally touching music enhances facial memory capacity. Sci. Rep..

[44] Näätänen R (2001). The perception of speech sounds by the human brain as reflected by the mismatch negativity (MMN) and its magnetic equivalent (MMNm). Psychophysiology.

[45] Escera C, Alho K, Winkler I, Näätänen R (1998). Neural mechanisms of involuntary attention to acoustic novelty and change. J. Cogn. Neurosci..

[46] Stupacher J, Wrede M, Vuust P (2022). A brief and efficient stimulus set to create the inverted U-shaped relationship between rhythmic complexity and the sensation of groove. PLoS ONE.

[47] Yerkes RM, Dodson JD (1908). The relation of strength of stimulus to rapidity of habit-formation. J. Comp. Neurol. Psychol..

[48] Brost, B., Mehrotra, R. & Jehan, T. The music streaming sessions dataset. In 2594–2600 (Association for Computing Machinery, 2019). 10.1145/3308558.3313641

[49] Heggli, O. A. Generalized spotify analyser. *GitHub Repos.* (2020).

[50] Spotify for Developers. https://developer.spotify.com/documentation/web-api/reference/object-model/ (2021).

[51] Dammann T, Haugh K (2017). Genre Classification of Spotify Songs Using Lyrics, Audio Previews, and Album Artwork.

[52] Rentfrow PJ, Gosling SD (2003). The do re mi’s of everyday life: The structure and personality correlates of music preferences. J. Pers. Soc. Psychol..

[53] Help—How do genre charts work?—Spotify for Artists. https://artists.spotify.com/help/article/how-genre-charts-work

[54] Nainggolan R, Perangin-angin R, Simarmata E, Tarigan AF (2019). Improved the performance of the K-means cluster using the sum of squared error (SSE) optimized by using the elbow method. J. Phys. Conf. Ser..

[55] Kestemont M-P (1987). The Kolmogorov distance as comparison measure between parametric and non-parametric Bayesian predictions. J. R. Stat. Soc. Ser. Stat..

[56] Allen M, Poggiali D, Whitaker K, Marshall TR, Kievit RA (2019). Raincloud plots: A multi-platform tool for robust data visualization. Wellcome Open Res..

[57] Dillman Carpentier FR, Potter RF (2007). Effects of music on physiological arousal: Explorations into tempo and genre. Media Psychol..

[58] Gomez P, Danuser B (2007). Relationships between musical structure and psychophysiological measures of emotion. Emotion.

[59] Loui P, Bachorik JP, Li HC, Schlaug G (2013). Effects of voice on emotional arousal. Front. Psychol..

[60] Cordi MJ, Ackermann S, Rasch B (2019). Effects of relaxing music on healthy sleep. Sci. Rep..

[61] Eysenck HJ (1963). Biological basis of personality. Nature.

[62] Huron D (2011). Why is sad music pleasurable? A possible role for prolactin. Music. Sci..

[63] Lee-Harris G, Timmers R, Humberstone N, Blackburn D (2018). Music for relaxation: A comparison across two age groups. J. Music Ther..

[64] Van Den Bosch, I., Salimpoor, V. & Zatorre, R. J. Familiarity mediates the relationship between emotional arousal and pleasure during music listening. *Front. Hum. Neurosci.* **7**, (2013). 10.3389/fnhum.2013.00534 PMC3763198 24046738

[65] Vuust P (2005). To musicians, the message is in the meter: Pre-attentive neuronal responses to incongruent rhythm are left-lateralized in musicians. Neuroimage.

[66] Vuust P, Brattico E, Seppänen M, Näätänen R, Tervaniemi M (2012). The sound of music: Differentiating musicians using a fast, musical multi-feature mismatch negativity paradigm. Neuropsychologia.

[67] Furnham A, Allass K (1999). The influence of musical distraction of varying complexity on the cognitive performance of extroverts and introverts. Eur. J. Personal..

